# CEREGLIDE 71 catheter for contact aspiration of secondary distal medium vessel occlusion: A case report

**DOI:** 10.1016/j.radcr.2026.03.014

**Published:** 2026-04-02

**Authors:** Tatsushi Hatayama, Hideki Endo, Koichiro Shindo, Daigo Goto, Michiru Katayama, Kazutaka Ikeda, Yasuyuki Tatsuta, Tomoaki Ishizuka, Masahiro Okuma, Ryunosuke Yoshihara, Daishi Yamaguchi, Yohei Yamaguchi, Suguru Sakurai, Tatsuya Ogino, Kentaro Fumoto, Koji Oka, Hirohiko Nakamura

**Affiliations:** a Department of Neurosurgery, Nakamura Memorial Hospital, South 1, West 14, Chuo-ku, Sapporo, Hokkaido 060-8570, Japan; b Department of Neurosurgery, Nakamura Memorial South Hospital, 2-2, Kawazoe, Minami-ku, Sapporo, Hokkaido 005-8555, Japan

**Keywords:** Acute ischemic stroke, Aspiration catheter, Contact aspiration, Distal medium vessel occlusion, Endovascular treatment, Mechanical thrombectomy

## Abstract

Mechanical thrombectomy for large vessel occlusion (LVO) in acute ischemic stroke may result in distal medium vessel occlusion (DMVO) during the procedure. Large-bore catheters are commonly selected for LVO treatment, but their use for DMVO is desirable from the perspective of simplifying the procedure and reducing procedure time. Here, we report a case of primary LVO with secondary DMVO successfully treated using a large-bore aspiration catheter (CEREGLIDE 71, 132 cm). To our knowledge, there are no previous reports describing the use of CEREGLIDE 71 not only for LVO but also for secondary DMVO treatment. A 74-year-old man with a history of atrial fibrillation was transported to our hospital by ambulance. Magnetic resonance imaging revealed acute cerebral infarcts, and magnetic resonance angiography demonstrated occlusion of the left internal carotid artery. Intravenous thrombolysis and mechanical thrombectomy were performed. A combined technique using CEREGLIDE 71 and a stent retriever achieved recanalization of the primary LVO but resulted in secondary DMVO (distal middle cerebral artery M2 occlusion). Under a modified working angle, the CEREGLIDE 71 used for LVO treatment was carefully advanced to the distal occlusion site. Contact aspiration with CEREGLIDE 71 achieved successful recanalization without complications. At 10 months after the procedure, there was no evidence of vascular injury. CEREGLIDE 71 is a large-bore aspiration catheter with high accessibility, allowing seamless transition to secondary DMVO treatment without device exchange. Careful device manipulation and detailed anatomical assessment of the vascular architecture leading to the occlusion site are required.

## Introduction

Mechanical thrombectomy (MT) for large vessel occlusion (LVO) in acute ischemic stroke (AIS) may result in distal medium vessel occlusion (DMVO) during the procedure, necessitating prompt transition to secondary DMVO treatment. CEREGLIDE 71 (CERENOVUS, Irvine, CA) is a large-bore aspiration catheter used in MT [1,2]. Large-bore catheters are commonly selected for LVO treatment, but their use as aspiration catheters for secondary DMVO would be highly desirable because it could simplify the procedure and reduce procedure time. To our knowledge, however, there are no previous reports describing the use of CEREGLIDE 71 not only for LVO but also for secondary DMVO treatment. We herein report a case of primary LVO with secondary DMVO successfully treated using the CEREGLIDE 71 aspiration catheter.

## Case report

**Fig. 1:**
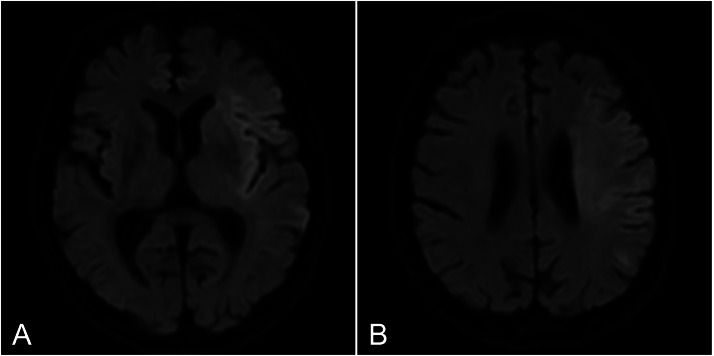
Magnetic resonance imaging findings at admission. (A, B) Diffusion-weighted images showing acute cerebral infarcts in the left middle cerebral artery territory.

The patient was a 74-year-old man with a history of hypertension, diabetes, and atrial fibrillation, for which anticoagulant therapy had been discontinued. He was found unconscious at his workplace and transported to our hospital by ambulance. On emergency arrival, he had severe neurological deficits, including impaired consciousness, aphasia, and right hemiplegia. His National Institutes of Health Stroke Scale score was 28. Electrocardiography showed atrial fibrillation. Magnetic resonance imaging at 127 minutes after last known well revealed acute cerebral infarcts in the left middle cerebral artery (MCA) territory (Fig. 1), and magnetic resonance angiography (MRA) showed occlusion of the left internal carotid artery (ICA).

**Fig. 2:**
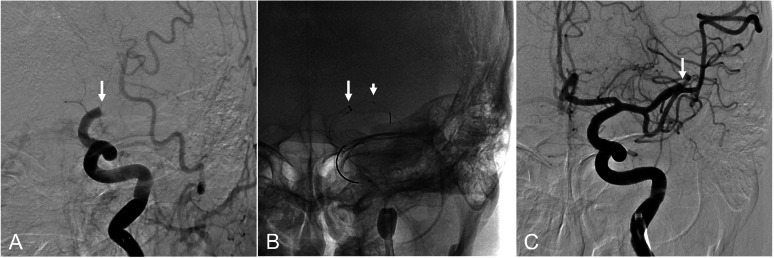
Mechanical thrombectomy for primary large vessel occlusion. (A) Digital subtraction angiography showing occlusion of the left intracranial internal carotid artery (arrow). (B) Combined technique using a CEREGLIDE 71 (132 cm) catheter (long arrow) and a stent retriever (short arrow). (C) Recanalization of the primary occlusion site with subsequent secondary distal medium vessel occlusion of the left middle cerebral artery M2 segment (arrow).

**Fig. 3:**
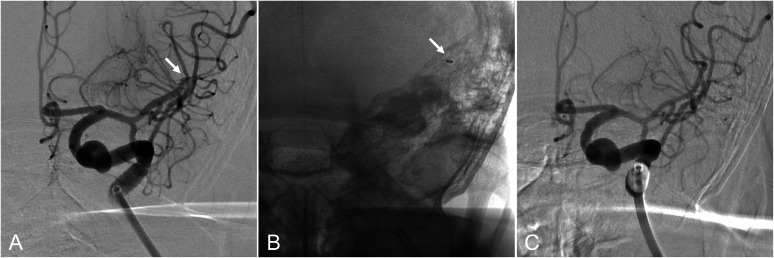
Mechanical thrombectomy for secondary distal medium vessel occlusion (anteroposterior view). (A) Modified working angle allowing improved visualization of the left middle cerebral artery M2 superior trunk leading to the occlusion site (arrow). (B) Contact aspiration using the CEREGLIDE 71 (132 cm) catheter (arrow). (C) Recanalization after mechanical thrombectomy.

**Fig. 4:**
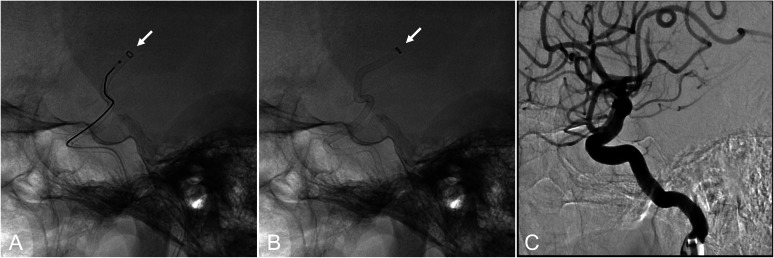
Mechanical thrombectomy for secondary distal medium vessel occlusion (lateral view). (A) Advancement of the CEREGLIDE 71 (132 cm) catheter to the occlusion site coaxially with a microcatheter (arrow). (B) Contact aspiration using the CEREGLIDE 71 catheter (arrow). Despite bends and vessel tortuosity during distal advancement, the CEREGLIDE 71 catheter remained intact without kinking and maintained luminal patency (C) Recanalization after mechanical thrombectomy.

Intravenous thrombolysis with recombinant tissue-type plasminogen activator was therefore initiated, followed by endovascular MT under local anesthesia without systematic heparinization (Fig. 2, Fig. 3, Fig. 4). The door-to-groin puncture time was 63 minutes. For the left intracranial ICA occlusion corresponding to LVO, MT was performed using a CEREGLIDE 71 (132 cm) in combination with an EmboTrap III 6.5 × 45 mm (CERENOVUS, Irvine, CA) (Fig. 2). The groin-to-first-pass time was 24 minutes. The primary LVO was successfully recanalized but subsequently transitioned to a distal occlusion of the left MCA M2 segment (secondary DMVO) (Fig. 2C).

**Fig. 5:**
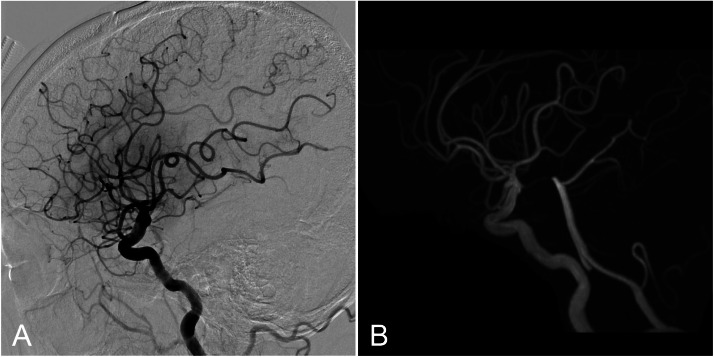
Postprocedural angiogram and follow-up magnetic resonance angiography (lateral view). (A) Postprocedural angiogram showing recanalization of primary large vessel occlusion and secondary distal medium vessel occlusion (modified Thrombolysis in Cerebral Infarction score of grade 2b). Final angiogram also demonstrating that no vessel injury occurred during advancement of the CEREGLIDE 71 (132 cm) catheter. (B) Follow-up magnetic resonance angiography showing recanalization of the residual occlusion without evidence of vascular injury.

The working angle was adjusted to improve visualization of the left MCA M2 superior trunk leading to the occlusion site (Fig. 3A). The same CEREGLIDE 71 used for LVO treatment was carefully advanced to the occlusion site, coaxially with a Phenom 21 (160 cm) microcatheter (Medtronic, Minneapolis, MN) (Figs. 3B, 4A and 4B). The groin-to-second-pass time was 36 minutes. Despite vessel bends and tortuosity during advancement into the distal segment, the CEREGLIDE 71 catheter remained intact without kinking and maintained luminal patency (Fig. 4B). Contact aspiration using CEREGLIDE 71 achieved successful recanalization of the secondary DMVO (Figs. 3C and 4C). The final modified Thrombolysis in Cerebral Infarction score was grade 2b (Fig. 5A). No procedure-related complications were observed.

Postoperatively, the patient received medical management, including anticoagulant therapy, and underwent rehabilitation. We started anticoagulant therapy (apixaban) two days after the procedure. Follow-up MRA showed recanalization of the residual occlusion (Fig. 5B). He was discharged home 2 months after stroke onset. At 3 months, his modified Rankin Scale score was 2. Follow-up MRA at 10 months after the procedure confirmed patency of the left MCA without evidence of vascular injury.

## Discussion

We have described a case of secondary DMVO successfully treated with contact aspiration using a CEREGLIDE 71 (132 cm) catheter. CEREGLIDE 71 is a variable-stiffness, single-lumen catheter with an inner diameter of 0.071 inches; its stiff proximal shaft transitions to a flexible distal shaft, facilitating advancement into a range of arterial anatomies [1]. Although endovascular MT for AIS is now well established and numerous devices are available, clinical studies focusing specifically on CEREGLIDE 71 remain limited [1,2]. A retrospective study evaluating the characteristics and technical outcomes of MT for AIS following the inaugural global use of the CEREGLIDE 71 intermediate catheter reported successful reperfusion in 88% of 25 patients at a single center, with a mean of 2.2 passes [1]. The CEREGLIDE 71 catheter has been optimized for aspiration (sufficient lumen size to generate high aspiration force), trackability, manipulability, support, and deliverability [1]. That study included four cases (16%) with MCA M2 occlusion classified as LVO, but did not include DMVO cases [1].

Given its distal outer diameter of 0.0820 inches (6.2 French), the CEREGLIDE 71 catheter may pose challenges for safe advancement into DMVO sites compared with lower-profile catheters. Nevertheless, in the present case, favorable recanalization of secondary DMVO was achieved using contact aspiration with CEREGLIDE 71 (Fig. 3, Fig. 4, Fig. 5). To the best of our knowledge, no previous reports have described the use of CEREGLIDE 71 not only for primary LVO but also for secondary DMVO treatment, as demonstrated here.

Recent studies have shown that MT for primary DMVO in AIS does not significantly improve functional outcomes or reduce mortality, while increasing the risk of hemorrhagic complications [[3], [4], [5], [6], [7]]. However, secondary DMVO differs from primary DMVO in its underlying pathophysiology, and data regarding its prognosis and optimal management remain limited. Compared with primary DMVO, secondary DMVO is often associated with more extensive ischemia and generally poorer outcomes [8,9]. Moreover, no randomized trials have compared treatment strategies for secondary DMVO. In this context, particularly when secondary DMVO occurs during MT for LVO, a more aggressive MT approach may be justified. Because vascular access has already been established, MT can be performed relatively quickly without additional access-related risks [9].

Careful device manipulation and detailed assessment of anatomical features, including vessel diameter and course, are essential in secondary DMVO treatment to minimize complications, especially intracranial hemorrhage. From a device-selection standpoint, using the same device for both LVO and secondary DMVO is desirable to simplify the procedure and reduce treatment time. The CEREGLIDE 71 catheter has a 55-cm hydrophilic coating near its distal soft tip; together with its relatively rigid proximal body, this design allows precise catheter control with a low risk of endothelial injury [1]. Although its distal outer diameter (0.0820 inches, 6.2 French) may be less favorable for distal navigation compared with lower-profile devices, we considered its use reasonable in selected cases because it enables rapid treatment of secondary DMVO without the need for device exchange. When appropriate, conversion to lower-profile devices or modification of procedural techniques may also be considered [10]. This strategy may additionally offer advantages from a health economics perspective. From a technical standpoint, adjustment of the working angle to better delineate the vascular anatomy leading to the occlusion site was helpful in this case (Fig. 3A). Although minor, this modification facilitated safe advancement of the large-bore CEREGLIDE 71 catheter to the distal occlusion site (Figs. 3B, 4A and 4B).

This study has two main limitations. First, it represents a single-institution case report, and larger studies are needed to assess generalizability. Second, long-term follow-up data are limited, and the possibility of delayed vascular injury cannot be fully excluded.

## Conclusion

We have presented a case demonstrating the utility of the CEREGLIDE 71 catheter not only for primary LVO but also for secondary DMVO. The large-bore aspiration catheter with good accessibility allows seamless transition to secondary DMVO treatment without device exchange. Careful device manipulation and thorough anatomical assessment of the vascular architecture leading to the occlusion site remain essential.

## Ethical statement

All procedures performed in studies involving human participants were in accordance with the ethical standards of the institution and/or national research committee and with the 1964 Helsinki declaration and its later amendments or comparable ethical standards. The study was approved by the Ethics Committee of Nakamura Memorial Hospital (No. 2026010701).

## CRediT author statement

**Tatsushi Hatayama:** Validation, Formal analysis, Investigation, Data curation, Writing - original draft, Writing - review & editing, Visualization. **Hideki Endo:** Conceptualization, Methodology, Validation, Formal analysis, Investigation, Resources, Data curation, Writing - original draft, Writing - review & editing, Visualization, Supervision, Project administration. **Koichiro Shindo:** Validation, Formal analysis, Investigation, Writing - review & editing. **Daigo Goto:** Validation, Formal analysis, Investigation, Resources, Writing - review & editing. **Michiru Katayama:** Validation, Formal analysis, Resources, Writing - review & editing. **Kazutaka Ikeda:** Validation, Formal analysis, Resources, Writing - review & editing. **Yasuyuki Tatsuta:** Validation, Writing - review & editing. **Tomoaki Ishizuka:** Validation, Writing - review & editing. **Masahiro Okuma:** Validation, Writing - review & editing. **Ryunosuke Yoshihara:** Validation, Writing - review & editing. **Daishi Yamaguchi:** Validation. **Yohei Yamaguchi:** Validation. **Suguru Sakurai:** Validation. **Tatsuya Ogino:** Validation. **Kentaro Fumoto:** Validation, Formal analysis, Resources, Writing - review & editing, Supervision. **Koji Oka:** Supervision. **Hirohiko Nakamura:** Supervision.

## Patient consent

Written informed consent was obtained from the patient.
